# Syndecan-1 Promotes Streptococcus pneumoniae Corneal Infection by Facilitating the Assembly of Adhesive Fibronectin Fibrils

**DOI:** 10.1128/mBio.01907-20

**Published:** 2020-12-08

**Authors:** Akiko Jinno, Atsuko Hayashida, Howard F. Jenkinson, Pyong Woo Park

**Affiliations:** a Department of Medicine, Boston Children’s Hospital, Boston, Massachusetts, USA; b Bristol Dental School, University of Bristol, Bristol, United Kingdom; c Department of Pediatrics, Harvard Medical School, Boston, Massachusetts, USA; University of Illinois at Chicago

**Keywords:** extracellular matrix, host-pathogen interactions, keratitis, proteoglycans, syndecans

## Abstract

Subversion of heparan sulfate proteoglycans (HSPGs) is thought to be a common virulence mechanism shared by many microbial pathogens. The prevailing assumption is that pathogens co-opt HSPGs as cell surface attachment receptors or as inhibitors of innate host defense. However, there are few data that clearly support this idea *in vivo*. We found that deletion of syndecan-1 (Sdc1), a major cell surface HSPG of epithelial cells, causes a gain of function in a mouse model of scarified corneal infection, where *Sdc1^−/−^* corneas were significantly less susceptible to Streptococcus pneumoniae infection. Administration of excess Sdc1 ectodomains significantly inhibited S. pneumoniae corneal infection, suggesting that Sdc1 promotes infection as a cell surface attachment receptor. However, S. pneumoniae did not interact with Sdc1 and Sdc1 was shed upon S. pneumoniae infection, indicating that Sdc1 does not directly support S. pneumoniae adhesion. Instead, Sdc1 promoted S. pneumoniae adhesion by driving the assembly of fibronectin (FN) fibrils in the corneal basement membrane to which S. pneumoniae attaches when infecting injured corneas. S. pneumoniae specifically bound to corneal FN via PavA, and PavA deletion significantly attenuated S. pneumoniae virulence in the cornea. Excess Sdc1 ectodomains inhibited S. pneumoniae corneal infection by binding to the Hep II domain and interfering with S. pneumoniae PavA binding to FN. These findings reveal a previously unknown virulence mechanism of S. pneumoniae where key extracellular matrix (ECM) interactions and structures that are essential for host cell homeostasis are exploited for bacterial pathogenesis.

## Abstract (precis)

Bacterial pathogens have evolved several ingenious mechanisms to subvert host cell biology for their pathogenesis. Bacterial attachment to the host ECM establishes a niche to grow and is considered one of the critical steps of infection. This pathogenic mechanism entails coordinated assembly of the ECM by the host to form the ECM structure and organization that are specifically recognized by bacteria for their adhesion. We serendipitously discovered that epithelial Sdc1 facilitates the assembly of FN fibrils in the corneal basement membrane and that this normal biological function of Sdc1 has detrimental consequences for the host in S. pneumoniae corneal infection. Our studies suggest that bacterial subversion of the host ECM is more complex than previously appreciated.

## INTRODUCTION

Microbial adhesion to host tissues is a key step in establishing a successful infection (1). A wide variety of pathogens bind to host extracellular matrix (ECM) components and their receptors for their attachment and invasion (2–7). For example, a large number of Gram-positive and Gram-negative bacteria bind specifically to fibronectin (FN) (8), one of the most abundant adhesive ECM proteins (9). So far, over 100 bacterial FN-binding proteins (FnBPs) have been identified (10). Similarly, many viruses, bacteria, and parasites interact specifically with the heparan sulfate (HS) motif of HS proteoglycans (HSPGs) (7, 11, 12), which are present ubiquitously on the surface of all adherent cells. Cell surface HSPGs function primarily as coreceptors for heparin-binding molecules, including many ECM components, by localizing and increasing the effective concentration of these molecules at the cell surface and facilitating their interaction with their respective signaling receptors (13, 14). The prevailing assumption is that microbial pathogens interact in a similar manner with cell surface HSPGs, primarily using them as the initial contact site with host cells. The majority of ECM components are inaccessible to pathogens in intact tissues but exposed upon injury to the epithelial barrier, which partly explains why tissue injury predisposes the host to infection.

However, while molecular and biochemical features of host ECM-pathogen interactions have been extensively studied, comparatively little is known about the contributions of these interactions to pathogenesis *in vivo*. In fact, both host cells and microbes are known to change their phenotypes when cultured *in vitro*, and some studies have suggested that the ability of microbes to interact with ECM components may be a result of cell culture adaptation (11, 15). Furthermore, studies comparing the virulence of wild-type (Wt) and ECM adhesin mutant strains have sometimes produced variable results (16). On the other hand, it is also true that a given pathogen can bind specifically to several ECM components and express several binding proteins for a particular ECM molecule. Whether this considerable overlap in interacting with the host ECM is the reason why some ECM adhesin mutant strains do not show significantly reduced virulence in animal studies is not known. Nonetheless, the fact that pathogens elaborate multiple mechanisms to interact with the host ECM suggests that these interactions are critical for pathogenesis. In addition, the seemingly redundant strategies to interact with the ECM might suggest that a particular ECM interaction is significant and relevant in only selecting host environments and niches, and in specific steps of pathogenesis.

The syndecan family of type I transmembrane HSPGs, comprised of four members in mammals (Sdc1 to -4), is the major source of cell surface HS (13). Syndecans are expressed on different cell types and locations at different times and levels and, hence, likely perform specific functions *in vivo*. For example, Sdc1 is predominantly expressed on the surface of epithelial cells, a cell type frequently targeted by microbes early in pathogenesis. Indeed, cell surface Sdc1 has been shown to promote Neisseria gonorrhoeae attachment and invasion of epithelial cells (17, 18). Sdc1 can also be shed from the cell surface as soluble HSPG ectodomains by metalloproteinases. Activation of Sdc1 shedding is an innate host response to tissue injury that protects against inflammatory tissue damage but can also be a pathogenic response that promotes infection by dampening inflammatory host defense when stimulated in an untimely manner (7). For example, Staphylococcus aureus and Pseudomonas aeruginosa induce Sdc1 shedding through specific virulence factors (19, 20) and exploit the ability of Sdc1 ectodomains to inhibit innate immune defense to enhance their survival *in vivo* (21–24).

The ability of HSPGs to support bacterial adhesion and to inhibit innate host defense is central to the pathogenesis of many microbes, and these functions, in principle, are potential therapeutic targets for many infectious diseases. Here, we examined the role of the syndecan family of HSPGs in Streptococcus pneumoniae corneal infection. Bacterial keratitis is a serious ocular surface infection that can lead to corneal opacity and loss of vision (25). Even with modern-day treatment, corneal infections can result in poor vision in 50% and surgical intervention in 12% of patients (26). Corneal injury, such as that caused by contact lens wear, trauma, and surgery, is a major factor that predisposes individuals to bacterial keratitis by exposing the ECM components and their receptors to pathogens for their attachment. Both Gram-positive and Gram-negative pathogens can cause bacterial keratitis, of which S. pneumoniae is one of the species frequently isolated from infected corneas (27–29). S. pneumoniae binds to HSPGs (30) and induces the shedding of Sdc1 ectodomains in cell-based assays (31). However, our studies show that cell surface Sdc1 is not an adhesion receptor for S. pneumoniae and Sdc1 ectodomains do not enhance S. pneumoniae virulence. Instead, our results reveal a new function of Sdc1 in bacterial pathogenesis whereby it facilitates S. pneumoniae adhesion to FN fibrils in the corneal basement membrane by driving FN matrix assembly.

## RESULTS

### Sdc1 is a major HSPG expressed in the corneal epithelium.

**FIG 1:**
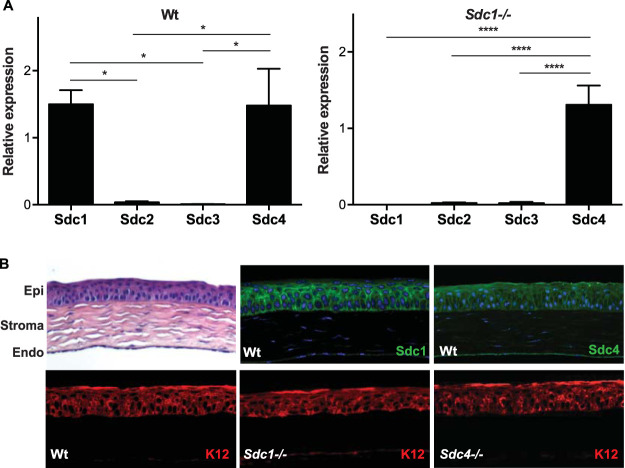
Sdc1 and Sdc4 are the major syndecans expressed in the mouse corneal epithelium. (A) Sdc1, Sdc2, Sdc3, and Sdc4 mRNA levels in unchallenged Wt and *Sdc1^−/−^* corneas were assessed by qRT-PCR. Δ*C_q_* values normalized to GAPDH were determined and shown as relative expression (mean ± SE, *n* = 4; ****, *P* < 0.0001; *, *P* < 0.05, ANOVA). (B) Hematoxylin and eosin (H&E)-stained Wt cornea section displaying the corneal epithelium (Epi), stroma, and endothelium (Endo); Wt cornea immunostained for Sdc1 or Sdc4; and Wt, *Sdc1^−/−^*, and *Sdc4^−/−^* corneas immunostained for K12 (original magnification, ×200).

We initially examined the expression of four syndecans in wild-type (Wt) mouse corneas by quantitative real-time PCR (qRT-PCR). Sdc1 and Sdc4 were strongly expressed, whereas Sdc2 and Sdc3 expression was minimal and approximately 50-fold lower than those of Sdc1 and Sdc4 (Fig. 1A). Consistent with the mRNA data, immunohistological analyses showed that Sdc1 is strongly and strictly expressed in the corneal epithelium, especially by cells of the wing and basal layers (Fig. 1B). Sdc1 was expressed over the entire surface of wing layer epithelial cells (Fig. 1B), an expression pattern of Sdc1 typical of stratified epithelial cells. Sdc1 expression in basal epithelial cells was predominantly on the basolateral surface (Fig. 1B), similar to the expression pattern of simple epithelial cells where Sdc1 is thought to function as an ECM and cell-cell adhesion receptor to stabilize the epithelial sheet. Sdc4 was expressed throughout the corneal epithelium, albeit at intensities markedly lower than those of Sdc1 (Fig. 1B). Sdc4 was also expressed weakly in the corneal endothelium. Both Sdc1 and Sdc4 were not detected in the corneal stroma. Very weak expression of Sdc2 was seen on keratocytes in the corneal stroma, whereas Sdc3 was not detected, corroborating the mRNA data. Knockout of Sdc1 or Sdc4 did not affect the expression of the corneal epithelial cell marker keratin 12 (K12) and compartmentalization of the corneal epithelium and stroma (Fig. 1B), suggesting that *Sdc1^−/−^* and *Sdc4^−/−^* corneas develop normally. Furthermore, mRNA levels of other syndecans were not increased in compensation for the loss of Sdc1 in *Sdc1^−/−^* corneas (Fig. 1A). These data indicate that Sdc1 and also Sdc4 to a lesser extent are the predominant syndecans expressed in the cornea.

### Sdc1 knockout is a gain-of-function mutation in S. pneumoniae corneal infection.

Based on the expression pattern of syndecans in the cornea, we next carried out experiments to determine if Sdc1 and Sdc4 modulate the pathogenesis of S. pneumoniae corneal infection. Several Gram-positive and Gram-negative pathogens, such as S. pneumoniae, S. aureus, and P. aeruginosa, secrete factors that specifically induce Sdc1 ectodomain shedding in cell-based assays. S. pneumoniae directly activates Sdc1 shedding via its metalloproteinase ZmpC (31), whereas S. aureus and P. aeruginosa stimulate Sdc1 shedding by host cells through the cytotoxin alpha-toxin (19) and metalloproteinase LasA (20), respectively. ZmpC is a virulence factor for S. pneumoniae lung infection in mice (32), and LasA is a virulence factor for P. aeruginosa corneal infection in mice (33), whereas S. aureus alpha-toxin functions as a virulence factor in many S. aureus diseases (34–36), including keratitis (37). Importantly, Sdc1 ectodomains enhance S. aureus virulence in mouse corneas (21, 22) and P. aeruginosa virulence in mouse lungs (23) and skin (24), suggesting that the ability to enhance Sdc1 shedding is an important virulence activity shared by several bacterial pathogens.

**FIG 2:**
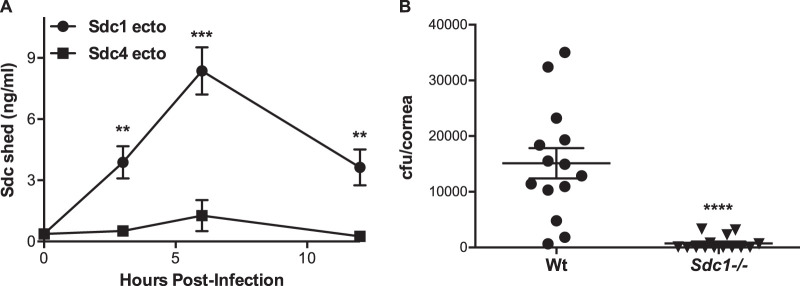
Sdc1 knockout is a gain-of-function mutation that enables mice to significantly resist S. pneumoniae corneal infection. (A) Injured Wt corneas were topically infected with 10^8^ CFU of S. pneumoniae TIGR4, and levels of Sdc1 and Sdc4 ectodomains in ocular surface fluids were measured at various times p.i. (mean ± SE, *n* = 6 for all groups; **, *P* < 0.01; ***, *P* < 0.001; 2-tailed *t* test). (B) Injured Wt and *Sdc1^−/−^* corneas were infected with 2 × 10^8^ CFU of TIGR4, and the corneal bacterial burden was measured at 6 h p.i. (*n* = 14 for both groups; ****, *P* < 0.0001, 2-tailed *t* test).

We therefore examined if S. pneumoniae also activates syndecan shedding *in vivo* and exploits the ability of shed ectodomains to enhance its virulence in the cornea. Intact cornea is highly resistant to bacterial infections, but it becomes susceptible to infection when the stratified epithelial barrier is breached. Wt mouse corneas were injured by 4 vertical scratches with a 26G needle and infected topically with 10^8^ CFU of S. pneumoniae TIGR4. At various times postinfection (p.i.), syndecan shedding was assessed by measuring Sdc1 and Sdc4 ectodomains in ocular surface fluids. Sdc1 ectodomains increased rapidly and significantly and reached a maximum at 20-fold over baseline at 6 h p.i., and high levels were sustained at 12 h p.i. (Fig. 2A). Injury alone did not induce Sdc1 shedding. On the other hand, Sdc4 ectodomains increased but were only slightly above background and significantly lower than Sdc1 ectodomains (Fig. 2A). Immunostaining of eye sections showed a markedly reduced expression of Sdc1, most prominently at the site of infection, whereas the signal for Sdc4 was unaffected (see Fig. S1A in the supplemental material), confirming the biochemical shedding data.

10.1128/mBio.01907-20.1 FIG S1 (A) Unchallenged and S. pneumoniae TIGR4 (10^8^ CFU)-infected Wt corneas were immunostained for Sdc1 and Sdc4 (original magnification, ×200). (B) Wt and *Sdc1^−/−^* corneas were infected with 4 × 10^8^ CFU of TIGR4, and the corneal bacterial burden was measured at the indicated times p.i. (mean ± SE, *n* = 8 for all groups; *, *P* < 0.05; **, *P* < 0.01). (C) Wt and *Sdc1^−/−^* corneas were infected with 3 × 10^8^ CFU of S. pneumoniae M11 or 10^8^ CFU of S. pneumoniae D39, and the corneal bacterial burden was measured at 6 h p.i. (*n* = 6 for all groups; **, *P* < 0.01; ***, *P* < 0.001). Download FIG S1, TIF file, 2.6 MB. Copyright © 2020 Jinno et al. 2020 Jinno et al. This content is distributed under the terms of the Creative Commons Attribution 4.0 International license.

To pursue if Sdc1 shedding is important in S. pneumoniae corneal pathogenesis, we initially compared the virulence of S. pneumoniae TIGR4 in Wt and *Sdc1^−/−^* corneas. Injured Wt and *Sdc1^−/−^* corneas were infected topically with TIGR4, and the corneal bacterial burden was measured at various times p.i. The bacterial burden was significantly reduced by over 19-fold at 6 h p.i. (Fig. 2B) and by 6-fold at 12 h p.i. (Fig. S1B) in *Sdc1^−/−^* corneas compared to Wt corneas infected identically. *Sdc1^−/−^* corneas were also significantly protected from infection by S. pneumoniae strains M11 and D39 (Fig. S1C), indicating that the ability of *Sdc1^−/−^* corneas to resist S. pneumoniae infection is not restricted to the TIGR4 strain. The prominent phenotype of *Sdc1^−/−^* corneas also indicated that Sdc4 and other HSPGs do not functionally compensate for the loss of Sdc1 in S. pneumoniae corneal infection. Altogether, these data suggest that Sdc1 is a host factor that prominently and specifically promotes S. pneumoniae corneal infection.

### Sdc1 does not promote S. pneumoniae corneal infection as an attachment receptor or as an inhibitor of host defense.

**FIG 3:**
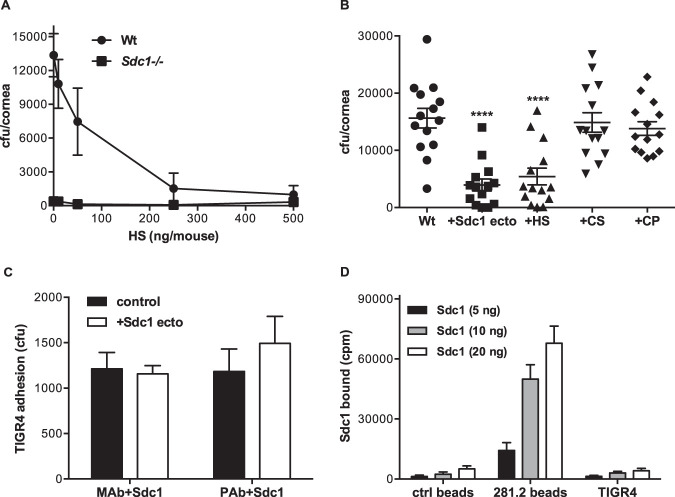
Excess Sdc1 ectodomain inhibits S. pneumoniae corneal infection, but Sdc1 is not an attachment receptor for S. pneumoniae. (A) Injured Wt and *Sdc1^−/−^* corneas were coinfected with 10^8^ CFU of S. pneumoniae TIGR4 and increasing doses of HS. The corneal bacterial burden was enumerated at 6 h p.i. (mean ± SE, *n* = 6 to 8). (B) Injured Wt corneas were coinfected with TIGR4 and 250 ng of purified Sdc1 ectodomain, HS, CS, or core protein (CP), and the corneal bacterial burden was measured at 6 h p.i. (mean ± SE, *n* = 14; ****, *P* < 0.0001, ANOVA). (C) Microtiter plates were coated with either 500 ng of 281.2 rat anti-mouse Sdc1 monoclonal or rabbit anti-mouse Sdc1 polyclonal antibodies and 1 μg of purified Sdc1 ectodomains. Plates were incubated with 10^5^ CFU of TIGR4 without (control) or with 50 μg/ml Sdc1 ectodomain (Sdc1 ecto). Attached bacteria were detached and quantified by plating out serial dilutions onto blood agar plates (mean ± SE, *n* = 5). (D) Increasing doses of radioiodinated Sdc1 ectodomains were incubated with 30 μl of affinity beads coupled without (ctrl beads) or with 281.2 anti-Sdc1 antibodies or with 5 × 10^8^ CFU of S. pneumoniae TIGR4 for 1 h at room temperature in PBS with 5% THY broth, and Sdc1 binding was assessed by counting bead- or bacterium-associated radioactivity (mean ± SE, *n* = 4).

Sdc1 ectodomains enhance S. aureus and P. aeruginosa virulence by inhibiting innate host defense in an HS-dependent manner (21–23). We tested if topical administration of HS similarly enhances S. pneumoniae virulence in *Sdc1^−/−^* corneas and found that it does not (Fig. 3A). Instead, we unexpectedly found that HS significantly inhibits S. pneumoniae infection in Wt corneas in a dose-dependent manner to the level of resistant *Sdc1^−/−^* corneas (Fig. 3A). Administration of purified Sdc1 ectodomains also significantly decreased the corneal bacterial burden in Wt corneas, but chondroitin sulfate (CS) and Sdc1 core protein devoid of HS and CS chains did not (Fig. 3B). These results indicate that instead of promoting infection, Sdc1 ectodomains inhibit S. pneumoniae corneal pathogenesis in an HS-dependent manner. Furthermore, neutrophil immunodepletion significantly increased the overall corneal bacterial burden in both Wt and *Sdc1^−/−^* mice, but induced neutropenia did not affect the significant difference in S. pneumoniae virulence seen in Wt and *Sdc1^−/−^* corneas (Fig. S2). These data suggest that functions of Sdc1 other than its immune modulating activities promote S. pneumoniae corneal infection.

10.1128/mBio.01907-20.2 FIG S2 Wt and *Sdc1^−/−^* mice were injected intravenously with 50 μg of rat anti-mouse Ly6G, and their corneal epithelium was injured 24 h later. Injured corneas were infected with 10^8^ CFU of S. pneumoniae TIGR4, and the corneal bacterial burden was measured at 6 h and 24 h p.i. (mean ± SE, *n* = 6; *, *P* < 0.05; **, *P* < 0.01). Download FIG S2, EPS file, 0.6 MB. Copyright © 2020 Jinno et al. 2020 Jinno et al. This content is distributed under the terms of the Creative Commons Attribution 4.0 International license.

We therefore tested the idea that S. pneumoniae co-opts Sdc1 as a cell surface attachment site to promote its infection. We first examined the ability of immobilized Sdc1 to support S. pneumoniae adhesion. Sdc1 was immobilized onto plates coated with either monoclonal or polyclonal anti-Sdc1 antibodies because Sdc1 does not bind well to plastic. S. pneumoniae attachment to immobilized Sdc1 was minimal and only slightly above background, and the low level of adhesion was not inhibited by addition of excess Sdc1 ectodomains (Fig. 3C). Furthermore, binding of increasing doses of radiolabeled Sdc1 ectodomains to S. pneumoniae TIGR4 was negligible compared to binding to affinity beads conjugated with 281.2 anti-Sdc1 antibodies and similar to background binding measured with unconjugated affinity beads (Fig. 3D). Radiolabeled Sdc1 ectodomains also did not bind to S. pneumoniae strains M11 and D39 with appreciable avidity (Fig. S3). Together, these observations indicate that S. pneumoniae does not interact directly with Sdc1 and does not use Sdc1 as an attachment receptor, which are consistent with the finding that Sdc1 is shed during S. pneumoniae infection.

10.1128/mBio.01907-20.3 FIG S3 Radioiodinated Sdc1 ectodomains (5 or 20 ng) were incubated with 30 μl of unconjugated affinity beads (ctrl beads) or with 5 × 10^8^ CFU of S. pneumoniae M11 or D39 for 1 h at room temperature in PBS with 5% THY broth, and Sdc1 binding was assessed by counting bead- or bacterium-associated radioactivity (mean ± SE, *n* = 4). Download FIG S3, EPS file, 0.6 MB. Copyright © 2020 Jinno et al. 2020 Jinno et al. This content is distributed under the terms of the Creative Commons Attribution 4.0 International license.

### Sdc1 drives the assembly of corneal basement membrane FN.

**FIG 4:**
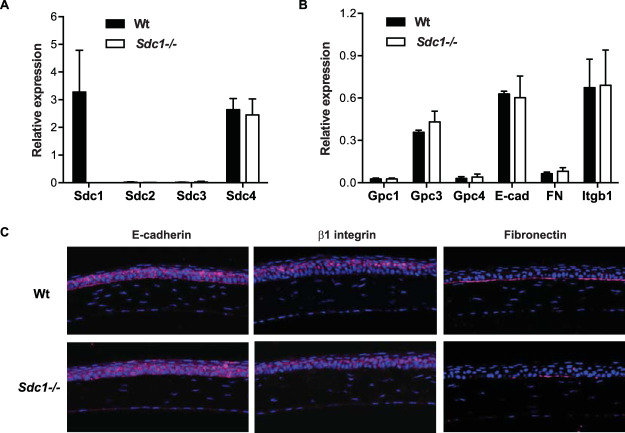
FN expression is markedly reduced in *Sdc1^−/−^* corneal basement membranes. (A and B) Sdc1, Sdc2, Sdc3, and Sdc4 mRNA expression (A) and Gpc1, Gpc3, Gpc4, E-cadherin (E-cad), FN, and β1 integrin (Itgb1) mRNA expression (B) in Wt and *Sdc1^−/−^* corneas at 6 h p.i. were assessed by qRT-PCR. Δ*C_q_* values normalized to GAPDH are shown as relative expression (mean ± SE, *n* = 4). (C) Wt and *Sdc1^−/−^* cornea sections were immunostained for E-cadherin, β1 integrin, and FN and counterstained with 4′,6-diamidino-2-phenylindole (DAPI) (original magnification, ×200).

Our results showed that HS chains of Sdc1 ectodomains inhibit S. pneumoniae infection, but cell surface Sdc1 does not support S. pneumoniae adhesion. We thus pursued the possibility that Sdc1 ectodomains inhibit S. pneumoniae corneal infection by interfering with bacterial binding to other HSPGs in Wt corneas and that these HSPG receptors are absent or expressed in reduced amounts in *Sdc1^−/−^* corneas. However, mRNA levels of other syndecans and also of glypican-1 (Gpc1), Gpc3, and Gpc4 were similar in infected Wt and *Sdc1^−/−^* corneas (Fig. 4A and B), which was confirmed by immunostaining (Fig. S4).

10.1128/mBio.01907-20.4 FIG S4 Wt and *Sdc1^−/−^* corneas were infected with 10^8^ CFU of S. pneumoniae TIGR4, and their corneas were isolated at 6 h p.i. Corneal sections were immunostained for Sdc4, Gpc1, or Gpc3 (original magnification, ×200). Download FIG S4, TIF file, 2.4 MB. Copyright © 2020 Jinno et al. 2020 Jinno et al. This content is distributed under the terms of the Creative Commons Attribution 4.0 International license.

Based on these data, we next explored the hypothesis that S. pneumoniae attaches to yet another ECM component when infecting injured corneas and that this interaction is inhibited by Sdc1 ectodomains or HS in Wt corneas and attenuated in *Sdc1^−/−^* corneas. Both Sdc1 and S. pneumoniae bind to several ECM components, suggesting that Sdc1 ectodomains may compete with S. pneumoniae for ECM receptor binding. Furthermore, Sdc1 regulates the expression and activity of integrins, E-cadherin, and FN, which are frequently exploited for microbial attachment. For example, Sdc1 colocalizes with E-cadherin and β1 integrin in cultured mammary gland epithelial cells, and Sdc1 knockdown significantly decreases the expression of E-cadherin and disrupts the polarized expression pattern of β1 integrins (38). Sdc1 also functions as a coreceptor for FN and regulates FN interactions with integrin receptors in epithelial and lymphoblastoid cells (39, 40).

Taking these findings into account, we examined if Sdc1 deletion reduces or dysregulates the expression of E-cadherin, β1 integrins, or FN in corneas, which in principle should decrease S. pneumoniae adhesion and infection in *Sdc1^−/−^* corneas. However, both infected Wt and *Sdc1^−/−^* corneas expressed similar levels of E-cadherin, β1 integrin, and FN mRNA (Fig. 4B). Immunostaining also showed that both E-cadherin and β1 integrin are similarly expressed in the epithelium of Wt and *Sdc1^−/−^* corneas (Fig. 4C), indicating that Sdc1 does not regulate the expression of E-cadherin and β1 integrin *in vivo*, at least in the cornea. On the other hand, immunostaining for FN unexpectedly showed that FN expression is substantially reduced in *Sdc1^−/−^* corneas compared to Wt corneas (Fig. 4C). FN was prominently and specifically expressed in the epithelial basement membrane in Wt mouse corneas, similar to the expression pattern of FN in human corneas (41, 42). However, FN expression was markedly decreased and punctuated in the basement membrane of *Sdc1^−/−^* corneas (Fig. 4C).

**FIG 5:**
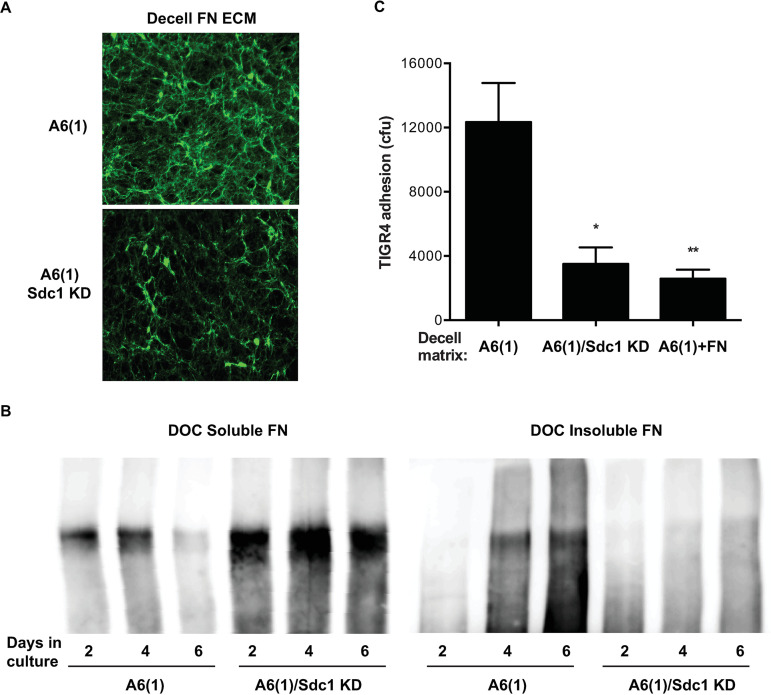
Sdc1 knockdown inhibits FN matrix assembly by corneal epithelial cells and attenuates S. pneumoniae attachment to the decellularized ECM. (A) One-day-postconfluent cultures of A6(1) cells transduced without or with LV-Sdc1 shRNA were decellularized, and the underlying FN matrix was assessed by immunostaining. (B) A6(1) cells transduced without or with LV-Sdc1 shRNA were seeded at 30% confluence and cultured for 2, 4, or 6 days. DOC soluble and insoluble fractions were separated by 7% SDS-PAGE and immunoblotted for FN. (C) The decellularized ECM of A6(1) cells and A6(1) cells transduced with LV-Sdc1 shRNA was incubated with 5 × 10^5^ CFU of S. pneumoniae in the absence or presence of 1 μg/ml FN, and bacterial attachment was enumerated 1 h later [mean ± SE, *n* = 5; *, *P* < 0.05, and **, *P* < 0.01, versus A6(1)].

The reduced expression of FN in *Sdc1^−/−^* corneal basement membranes, despite similar levels of FN mRNA in Wt and *Sdc1^−/−^* corneas, suggested that Sdc1 promotes the assembly of FN fibrils in the corneal basement membrane. We pursued this idea by testing if Sdc1 knockdown similarly inhibits FN matrix assembly by cultured corneal epithelial cells. While both epithelial and stromal cells are capable of synthesizing and assembling FN, Sdc1 is strictly expressed in the corneal epithelium, suggesting that Sdc1 primarily regulates FN assembly by epithelial cells. Transduction of A6(1) mouse corneal epithelial cells with lentivirus harboring Sdc1 short hairpin RNA (shRNA) (LV-Sdc1 shRNA) reduced cell surface Sdc1 expression by approximately 85% (Fig. S4) but did not affect FN mRNA levels. Confluent cultures of A6(1) cells treated without or with LV-Sdc1 shRNA were decellularized, and the remaining ECM was immunostained for FN. Decellularization does not affect the organization of the underlying ECM (43). Consistent with the *in vivo* findings, FN assembly by A6(1) cells treated with LV-Sdc1 shRNA was markedly decreased compared to that of untreated A6(1) cells that made robust FN fibrils (Fig. 5A).

The importance of Sdc1 in FN fibrillogenesis was also assessed by the deoxycholate (DOC) solubility assay, where a stable FN matrix could be distinguished by insolubility in 2% DOC (44). The proportion of DOC-insoluble FN increased in a time-dependent manner in A6(1) cells, but it did not increase in A6(1) cells treated with LV-Sdc1 shRNA (Fig. 5B). In fact, the majority of FN in Sdc1 knockdown A6(1) cells remained DOC soluble (Fig. 5B), indicating that they were not assembled into stable FN fibrils. Importantly, we found that S. pneumoniae adhesion onto the decellularized ECM produced by Sdc1-knockdown A6(1) cells was significantly decreased compared to adhesion onto the ECM produced by control A6(1) cells (Fig. 5C). Furthermore, excess FN significantly inhibited S. pneumoniae adhesion onto the decellularized ECM produced by untreated A6(1) cells (Fig. 5C). These results indicate that Sdc1 drives the assembly of FN in corneal basement membranes and that this biological function of Sdc1 generates the adhesive FN fibrils required for S. pneumoniae attachment.

### S. pneumoniae attaches to FN to infect injured corneas.

**FIG 6:**
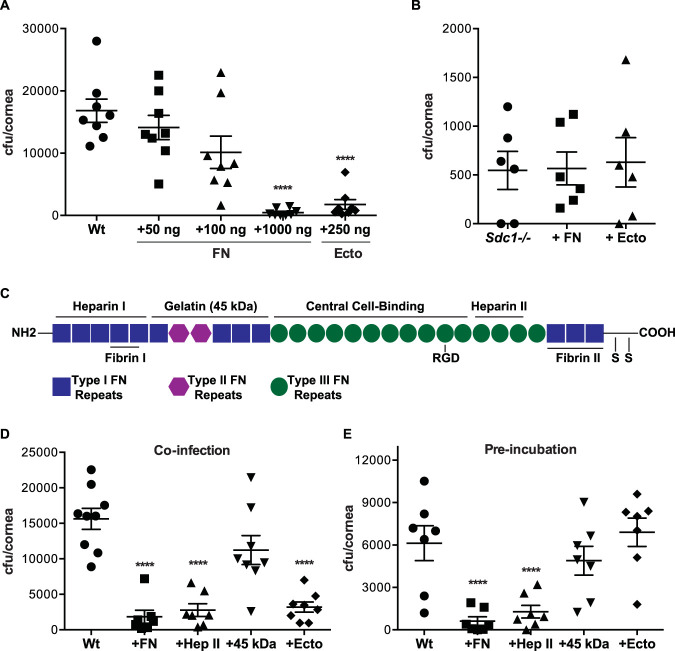
S. pneumoniae attaches to the Hep II domain of FN when infecting injured corneas. (A and B) Injured Wt (A) and *Sdc1^−/−^* (B) corneas were infected with 2 × 10^8^ CFU S. pneumoniae TIGR4 or coinfected with TIGR4 and FN or Sdc1 ectodomain as indicated. The corneal bacterial burden was measured at 6 h p.i. (mean ± SE, *n* = 8 in panel A, *n* = 6 in panel B). (In panel A, Wt versus 1,000 ng FN, ****, *P* < 0.0001; Wt versus 250 ng Ecto, ****, *P* < 0.0001). (C) Schematic diagram of FN domains. (D) Injured Wt corneas were infected with 2 × 10^8^ CFU TIGR4 or coinfected with TIGR4 and 1,000 ng FN, Hep II heparin-binding fragment, or 45-kDa gelatin binding fragment, or 250 ng Sdc1 ectodomain, and the corneal bacterial burden was measured at 6 h p.i. (*n* = 7 to 9; ****, *P* < 0.0001). (E) TIGR4 (10^8^ CFU) was preincubated with vehicle, 1,000 ng FN, Hep II fragment, or 45-kDa fragment, or 250 ng Sdc1 ectodomain for 30 min; washed; and topically applied to injured Wt corneas. The corneal bacterial burden was assessed at 6 h p.i. (*n* = 7; ****, *P* < 0.0001).

To pursue the importance of FN binding *in vivo*, Wt corneas were coinfected with S. pneumoniae and increasing doses of FN. Administration of FN significantly inhibited S. pneumoniae corneal infection in a dose-dependent manner, and at the highest dose tested, FN had a similar inhibitory effect as that of purified Sdc1 ectodomains where the corneal bacterial burden was decreased by more than 95% compared to control (Fig. 6A). Neither coinfection with excess FN nor Sdc1 ectodomain affected the low level of S. pneumoniae infection in *Sdc1^−/−^* corneas (Fig. 6B).

We next compared the effects of FN domain fragments on S. pneumoniae corneal infection. FN, initially discovered as a nonintegral protein on the surface of transformed cells (45), is a large multidomain protein that contains binding sites for integrins, ECM components, and coagulation factors, among others (9). Notably, FN contains 2 major heparin/HS binding domains in the N terminus (Hep I) and toward the C terminus following the cell-binding domain (Hep II) (Fig. 6C). When coinfected with S. pneumoniae, similar to the effects of intact FN and Sdc1 ectodomains, the 30-kDa Hep II fragment significantly decreased the corneal bacterial burden by 82%, whereas coinfection with the 45-kDa gelatin binding fragment did not (Fig. 6D). However, when the test compounds were preincubated with bacteria and removed prior to infection, intact FN and Hep II fragments significantly inhibited S. pneumoniae corneal infection, but Sdc1 ectodomains did not (Fig. 6E). Furthermore, binding of Sdc1 ectodomains to Hep II fragments conjugated to affinity beads was inhibited by excess Hep II fragments in a dose-dependent manner but not by the 45-kDa gelatin binding fragment (Fig. S6). Consistent with the finding that S. pneumoniae specifically binds to the C-terminal heparin-binding domain in FN (2), these results suggest that S. pneumoniae attaches to the Hep II domain of FN when infecting injured corneas. These data also indicate that excess Sdc1 ectodomains and HS inhibit S. pneumoniae corneal infection by binding to the Hep II domain and blocking S. pneumoniae attachment to FN.

10.1128/mBio.01907-20.5 FIG S5 A6(1) mouse corneal epithelial cells were transduced without or with LV-Sdc1 shRNA. The efficiency of Sdc1 knockdown was assessed by immunostaining (A) and quantification (B) of cell surface Sdc1 by mild trypsinization and dot immunoblotting (mean ± SE, *n* = 3). Download FIG S5, TIF file, 2.9 MB. Copyright © 2020 Jinno et al. 2020 Jinno et al. This content is distributed under the terms of the Creative Commons Attribution 4.0 International license.

10.1128/mBio.01907-20.6 FIG S6 Sdc1 (50 ng) was preincubated with PBS or increasing doses of Hep II or 45-kDa gelatin binding fragment as indicated for 30 min at room temperature, incubated overnight with 10 μl of Hep II-conjugated Ultralink beads at 4°C, and washed with PBS. Bound Sdc1 was eluted with 7 M urea/TBS and quantified by dot immunoblotting (mean ± SE, *n* = 3; ****, *P* < 0.0001). Download FIG S6, EPS file, 0.6 MB. Copyright © 2020 Jinno et al. 2020 Jinno et al. This content is distributed under the terms of the Creative Commons Attribution 4.0 International license.

### PavA is a virulence factor for S. pneumoniae corneal infection.

We next examined how S. pneumoniae binds to the Hep II domain of FN by testing the effects of pneumococcal adherence and virulence factor A (PavA) mutation on S. pneumoniae corneal infection. S. pneumoniae expresses several FN-binding proteins (FnBPs) (6, 46), of which PavA and PavB have been shown to bind to the C-terminal Hep II domain but not to the N-terminal Hep I domain of FN (47). PavA mutation attenuates S. pneumoniae virulence in mouse models of sepsis (48), pneumonia (49), and meningitis (50), but the role of PavA in corneal infection is unknown. PavA is found in all S. pneumoniae strains examined, yet its deletion does not affect bacterial growth and morphology (49), suggesting that the biological function of PavA is strictly to enhance S. pneumoniae virulence.

**FIG 7:**
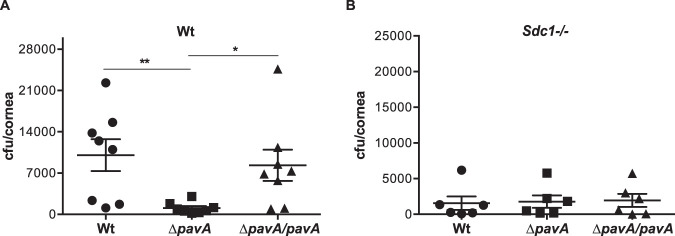
PavA is a virulence factor for S. pneumoniae corneal infection. Injured Wt (A) and *Sdc1^−/−^* (B) corneas were infected with 3 × 10^8^ CFU Wt S. pneumoniae R800, mutant R800 lacking *pavA* (Δ*pavA*), or Δ*pavA* mutant complemented with Wt *pavA* (Δ*pavA/pavA*). The corneal bacterial burden was enumerated at 6 h p.i. (*n* = 8 for panel A; **, *P* < 0.01; *, *P* < 0.05; *n* = 6 for panel B).

Wt and *Sdc1^−/−^* corneas were infected with Wt S. pneumoniae R800, mutant R800 lacking *pavA* (Δ*pavA*), or a Δ*pavA* mutant complemented with a wild-type copy of *pavA* (Δ*pavA/pavA*). Compared to Wt corneas infected with S. pneumoniae R800, the bacterial burden in Wt corneas infected with the Δ*pavA* strain was significantly decreased by 88%, whereas the bacterial burden in Wt corneas infected with the Δ*pavA/pavA* strain was similar to that of control (Fig. 7A). The virulence of R800 in *Sdc1^−/−^* corneas was not affected by *pavA* deletion or complementation (Fig. 7B). Together, these data indicate that PavA-mediated FN binding is a major virulence activity for S. pneumoniae corneal infection and suggest that blocking S. pneumoniae adherence to FN fibrils in the corneal basement membrane may be a viable therapeutic option for S. pneumoniae keratitis.

## DISCUSSION

Our studies on the role of syndecans in S. pneumoniae corneal infection showed that Sdc1 knockout causes a specific gain of function that enables mice to significantly resist infection. Previous studies have shown that Sdc1 can promote bacterial infection as a cell surface attachment receptor or as a soluble ectodomain inhibitor of innate host defense. However, our present studies show that neither of these Sdc1 functions enhances S. pneumoniae virulence at the ocular surface. Instead, the molecular basis of these unexpected findings was traced to a new role for Sdc1 in driving FN matrix assembly in the corneal basement membrane and promoting the formation of adhesive FN fibrils required for S. pneumoniae attachment.

Our results indicate that S. pneumoniae does not target Sdc1 or other HSPGs for its attachment in the cornea, despite the fact that S. pneumoniae binds to HS. The reason for this observation is not understood. The fine structure of HS dictates how HSPGs interact with their ligands. For example, 2-*O*-sulfated HS motifs are important in HSPG binding to FN (51). HS fine structures elaborated by HSPGs are also thought to be unique among HSPGs expressed by different cells. While the essential fine structure of HS that is recognized by S. pneumoniae is not known, these features suggest that corneal HSPGs, including Sdc1, might not contain the HS motifs required for S. pneumoniae binding. Consistent with this idea, although many bacteria can bind to HS, not all HS-binding bacteria bind to native HSPGs (22, 23).

Several criteria also indicate that S. pneumoniae does not exploit the ability of Sdc1 ectodomains to inhibit innate immune defense for its pathogenesis, despite the fact that S. pneumoniae specifically stimulates Sdc1 shedding both in cell-based assays and *in vivo* from the surface of corneal epithelial cells. The fact that the concentration of endogenous Sdc1 ectodomains shed during S. pneumoniae infection is several-hundredfold lower than that of the exogenously added Sdc1 ectodomains or HS likely explains why the naturally shed Sdc1 ectodomains are unable to inhibit S. pneumoniae infection in Wt corneas. However, the question still remains why S. pneumoniae specifically induces Sdc1 shedding when infecting the cornea. Because Sdc1 binds specifically to the C-terminal heparin binding domain in FN with high affinity (39), Sdc1 on the basal surface of basal epithelial cells is likely tightly bound to the region in basement membrane FN where S. pneumoniae attaches. These observations suggest that S. pneumoniae may induce Sdc1 shedding to detach Sdc1 from FN and unmask the adhesive Hep II binding domain in FN. Additional studies with specific inhibitors of Sdc1 shedding or with a mutant S. pneumoniae strain deficient in its ability to shed Sdc1 ectodomains are needed to test these hypotheses. Regardless, our results indicate that there is no direct role for cell surface or shed Sdc1 in promoting S. pneumoniae corneal infection.

Functions of Sdc1 in the stratified epithelium are largely unknown. Furthermore, while one of the first identified functions of Sdc1 was to bind to fibrillar ECM components, such as interstitial collagens (52) and FN (39), the role of Sdc1 in regulating ECM assembly is incompletely understood. Our studies identify for the first time that epithelial Sdc1 drives FN fibrillogenesis in the corneal basement membrane, but precisely how this is accomplished remains to be determined. Soluble FN is assembled into FN fibrils in a cell-mediated process that involves FN-FN interactions, integrins, and cell surface HSPGs (53, 54). CHO cells lacking HS do not assemble an FN matrix (55), indicating that HSPGs are important cofactors. In fact, Sdc2, but not Sdc4, has been shown to enhance FN assembly in cultured mesenchymal cells (56, 57). HS is thought to promote the assembly of FN into detergent-insoluble fibrils by bringing together soluble FN and promoting FN-FN interactions in the N-terminal type I modules (55, 58). Based on these findings, we propose that corneal epithelial Sdc1 promotes FN fibrillogenesis by binding to the Hep II domain via its HS chains and bringing FN monomers closer to facilitate FN-FN interactions in the N terminus.

Our studies show that S. pneumoniae binds to FN in the corneal basement membrane through PavA and that this is a major virulence mechanism for S. pneumoniae corneal infection. However, S. pneumoniae expresses at least 4 other FnBPs on its cell surface (47). The reason for the predominant role of PavA in S. pneumoniae corneal infection, despite this plasticity for FN binding, is not clearly understood. Having multiple means to bind to a major adhesive protein like FN might allow S. pneumoniae to adhere to and infect multiple tissues, such as the epithelium of upper and lower respiratory tracts, meninges, middle ear, and cornea (59). Consistent with this idea, mutation of each FnBP reduces virulence in different models of S. pneumoniae infection, suggesting a level of specificity for each FnBP in select niches.

Alternatively, the specific functions of S. pneumoniae FnBPs may be controlled by the expression pattern and the avidity of each FnBP for specific FN structures. S. pneumoniae FnBPs may be differentially regulated in certain host environments so that each comes into action only when needed. FN fibrils made by different cell types may also have unique structural features that are preferentially recognized by certain S. pneumoniae FnBPs. For example, normal and pathological cells assemble FN fibrils with different structures and functions (60). The fine structure and associated components of FN matrices are also likely to be different in different tissues, for example, when assembled in basement membranes and in interstitial ECMs.

Progression to fulminant infection requires additional events after the initial attachment to host tissues. PavA-mediated S. pneumoniae adhesion onto FN might trigger subsequent virulence activities during its pathogenesis in the cornea. The finding that PavA can modulate the expression and function of other virulence factors in S. pneumoniae meningitis (50) is consistent with this idea. In addition, FN fibrillogenesis and ECM reassembly are crucial steps of a normal tissue repair response. Hence, S. pneumoniae attachment to the Hep II domain of FN via PavA may interfere with HSPG-facilitated FN-FN interactions and reassembly of the FN matrix, which are expected to delay tissue repair and further promote pathogenesis. Our results support the concept that host-pathogen interactions are dynamic and that bacterial attachment is one of the key steps of the infectious cascade that is mechanistically linked to other virulence activities.

## MATERIALS AND METHODS

### Materials.

Todd-Hewitt broth, yeast extract, Epilife culture medium, fetal bovine serum (FBS), human corneal growth supplement, nitrocellulose membranes, and the Verso 1-step RT-qPCR kit were purchased from Thermo Fisher (Waltham, MA). Trypticase soy agar (TSA) plates with 5% sheep blood were from BD Biosciences (San Jose, CA). Immobilon Ny+ was from Millipore (Bedford, MA). Porcine mucosal HS was from Neoparin (Alameda, CA). Recombinant mouse Sdc1 ectodomains devoid of HS and CS were expressed as a glutathione *S*-transferase (GST) fusion protein in Escherichia coli and purified by glutathione affinity chromatography (23). Sodium deoxycholate (DOC), *N*-acetylcysteine (NAC), bovine tracheal CS-A, human fibronectin, and the 45-kDa gelatin-binding fragment of fibronectin were purchased from Sigma (St. Louis, MO). Sdc1 Mission shRNA lentiviral transduction particles (TRCN0000302270) and lentiviral packaging mix were from Sigma and used to generate lentivirus-harboring shRNA against mouse Sdc1 (LV-Sdc1 shRNA) in 293FT cells. Oligonucleotide primers were from IDT (Coralville, IA). The RNeasy Plus minikit was from Qiagen (Germantown, MD). Native Sdc1 ectodomains were purified from the conditioned medium of normal murine mammary gland (NMuMG) epithelial cells by DEAE chromatography, CsCl density centrifugation, and immunoaffinity chromatography (22, 61). The 30-kDa fibronectin fragment containing the Hep II domain was generated by thermolysin digestion of intact fibronectin and purified as described previously (62). All other materials except for the immunochemicals were purchased from Thermo Fisher, Sigma, or VWR.

### Immunochemicals.

Monoclonal rat anti-mouse Sdc1 ectodomain (281.2, 5 μg/ml for immunocytochemistry [ICC] and immunohistochemistry [IHC]), rat anti-mouse Ly6G (1A8, 50 μg/mouse for immunodepletion), and hamster anti-mouse β1 integrin (HMβ1-1, 5 μg/ml for IHC) antibodies were purchased from BioLegend (San Diego, CA). Monoclonal rat anti-mouse Sdc4 ectodomain antibodies (Ky8.2, 5 μg/ml for IHC) were from BD Biosciences. Polyclonal rabbit anti-mouse Sdc2 (MSE-2, 2 μg/ml for IHC) and anti-mouse Sdc3 (MSE-3, 5 μg/ml for ICC and IHC) antibodies were generated as described previously (63). Rabbit anti-recombinant human Sdc1 ectodomain antibodies, which cross-react with mouse Sdc1 (5 μg/ml for ICC and IHC), were generated in-house. Affinity-purified goat anti-mouse E-cadherin antibodies (2 μg/ml for IHC) were from R&D Systems (Minneapolis, MN). Mouse antifibronectin (FN-15, 5 μg/ml for ICC and IHC) and anti-HS (T320.11, 5 μg/ml for IHC) antibodies were from Sigma. Rabbit anti-Gpc1 and goat anti-Gpc3 antibodies (10 μg/ml for IHC) were from Bioss (Woburn, MA) and Santa Cruz (Dallas, TX), respectively. Rabbit anti-mouse keratin 12 antibodies (1 μg/ml for IHC) were a gift from Winston Kao (University of Cincinnati). Conjugated secondary antibodies were purchased from Jackson ImmunoResearch (West Grove, PA) or BioLegend.

### Bacteria.

Wild-type S. pneumoniae strains TIGR4, D39, M11, and R800, and the isogenic mutant R800 strain lacking the gene for *pavA* (Δ*pavA*) and the Δ*pavA* strain complemented with a wild-type copy of *pavA* (Δ*pavA/pavA*) were from our culture collection (31, 49). S. pneumoniae strains were grown on 5% sheep blood TSA plates or in 4 ml of Todd-Hewitt broth supplemented with 0.5% yeast extract (THY broth) in glass tubes without agitation to mid-log growth phase. The bacterial concentration was approximated by measuring absorbance at an optical density at 600 nm (OD_600_). After washing, the concentration was adjusted to the desired concentration for the *in vitro* binding assays and *in vivo* corneal infection assays as indicated. Viable S. pneumoniae was enumerated by colony formation on 5% sheep blood TSA plates to determine the exact infectious inoculum.

### Mice.

Unchallenged *Sdc1^−/−^* mice on the BALB/c background are healthy with normal growth, reproduction, tissue morphology, complete blood cell counts, and serum chemistry parameters. Both female and male *Sdc1^−/−^* mice and corresponding Wt littermates were used at an age of 8 to 12 weeks. Mice were maintained in microisolator cages under specific-pathogen-free conditions in a 12-h light/dark cycle and fed a basal rodent chow *ad libitum*. All animal experiments were approved by the Institutional Biosafety Committee (IBC) and Institutional Animal Care and Use Committee (IACUC) of Boston Children’s Hospital and complied with federal guidelines for research with experimental animals.

### S. pneumoniae corneal infection.

Four vertical scratches were made with a 26G by 3/8-in. needle in one of the corneas of each anesthetized mouse without penetrating beyond the superficial stroma. Various doses of S. pneumoniae strains in 5 μl phosphate-buffered saline (PBS) without or with test reagents were applied topically to the injured corneas. At the indicated times p.i., mice were euthanized, and eyes were enucleated and transected posterior to the corneal limbus under a dissecting microscope. The bacterial burden in isolated corneas or in whole enucleated eyes was determined by homogenizing in THY containing 0.1% (vol/vol) Triton X-100, plating out serial dilutions onto blood agar plates, and counting the number of CFU. In some experiments, S. pneumoniae bacteria were preincubated with test reagents for 1 h at room temperature, washed to remove unbound reagents, and applied topically to injured corneas.

### Sdc1 shedding.

Infected corneas of euthanized mice were incubated with 5 μl of 1% NAC in PBS for 5 min to break the mucous layer of tear film and facilitate recovery of ocular surface fluids (64). The ocular surface was then rinsed with 5 μl of 1% NAC and 10 μl of PBS. The collected ocular surface fluids were combined and spun down, and the concentration of Sdc1 and Sdc4 ectodomains was determined by dot immunoblotting (19).

### Sdc1-S. pneumoniae binding interaction.

In the soluble-phase binding assay, 5 μg of purified Sdc1 ectodomain was radioiodinated with 0.5 mCi of Na^125^I using the Bolton-Hunter reagent (22). Increasing doses of radioiodinated Sdc1 (5 to 20 ng) were incubated with 30 μl CNBr-activated Sepharose 4B beads inactivated with 1 M ethanolamine, 30 μl CNBr-activated Sepharose 4B beads conjugated with 281.2 anti-Sdc1 antibodies, or 5 × 10^8^ CFU S. pneumoniae TIGR4, M11, or D39 for 1 h at room temperature in 200 μl of THY broth. After washing, bound Sdc1 was quantified by measuring bead- or bacterium-associated radioactivity in a gamma counter. In the solid-phase binding assay, high-antibody-binding 96-well microplates were coated with 500 ng of either anti-Sdc1 ectodomain monoclonal or polyclonal antibodies. After washing away unbound antibodies, wells were incubated with 1 μg of purified native Sdc1 ectodomain and blocked with 1% bovine serum albumin (BSA). S. pneumoniae TIGR4 (10^5^ CFU) was added without or with 5 μg of Sdc1 ectodomains and incubated for 2 h at room temperature. Attached bacteria were detached by consecutive incubations in 100 μg/ml trypsin and THY broth containing 0.1% Triton X-100 and quantified by plating out serial dilutions onto blood agar plates.

### Sdc1 knockdown.

Mouse A6(1) corneal epithelial cells were a gift from Joram Piatigorsky (NIH/NEI). A6(1) cells were cultured in Epilife medium containing 10% FBS and 1× human corneal growth supplement. Confluent A6(1) cells were infected with LV-Sdc1 shRNA for 48 h. A6(1) cells were then washed and cultured for 24 h in 4 μg/ml puromycin. Knockdown efficiency was quantified by mild trypsinization of surface proteins and dot immunoblotting (19) and by fluorescence-activated cell sorting (FACS) analysis.

### Fibronectin fibrillogenesis.

A6(1) cells transduced without or with LV-Sdc1 shRNA were seeded at 30% confluence in 8-well chamber slides and cultured for 6 days. Cells were washed twice with PBS and three times with 100 mM Na_2_HPO_4_, pH 9.6, containing 2 mM MgCl_2_ and 2 mM EGTA. Cells were then lysed by incubating twice in 8 mM Na_2_HPO_4_, pH 9.6, containing 1% NP-40 for 15 min each at 37°C. The decellularized ECM was washed three times with 10 mM Na_2_HPO_4_, pH 7.5, containing 300 mM KCl, and then washed five times with deionized H_2_O. The decellularized ECM was fixed with 4% formaldehyde, blocked, and immunostained with antifibronectin monoclonal antibodies and Alexa 488 secondary antibodies. Images were captured with a Zeiss Axiovert 40 CFL microscope and AxioCam high-resolution camera.

### S. pneumoniae adhesion.

S. pneumoniae TIGR4 (10^5^ CFU) in PBS containing 5% (vol/vol) THY broth was added to the decellularized ECM assembled by A6(1) cells or A6(1) cells transduced with LV-Sdc1 shRNA in 24-well plates and incubated for 1 h at 37°C without or with 10 μg/ml fibronectin. Unbound bacteria were removed by washing 3 times with PBS, and bound bacteria were recovered by incubating with 0.1% Triton X-100/THY broth for 30 min at room temperature. Serial dilutions were plated onto blood agar plates to determine S. pneumoniae adhesion.

### DOC solubility.

Cells were seeded at 30% confluence in 6-well plates and cultured for 2, 4, or 6 days. Cells were washed twice with ice-cold PBS, and 200 μl of ice-cold DOC buffer (2% DOC, protease inhibitor cocktail, and 2 mM EDTA in 20 mM Tris, pH 8.8) was added. Cells were detached with a cell scraper, transferred to microcentrifuge tubes, sonicated, incubated for 30 min at 4°C with agitation, and centrifuged at 16,000 × *g* for 45 min at 4°C. After collecting the supernatant (DOC soluble fraction), 20 μl of lysis buffer (1% SDS, protease inhibitor cocktail, 2 mM EDTA in 40 mM Tris, pH 8.8) was added to the cell pellet and boiled for 1 min. Twenty microliters of 2× SDS sample buffer was then added and boiled for another 5 min (DOC insoluble fraction). Both DOC soluble and insoluble fractions were separated by 7% SDS-PAGE and subjected to Western blot analysis for fibronectin. Sample loading volume was normalized to band intensities of β-actin.

### Quantitative real-time PCR.

Corneas were dissected from enucleated eyes under a stereomicroscope, and RNA was isolated using the RNeasy Plus minikit. RNA concentration was determined with NanoDrop Lite (Thermo Fisher). qRT-PCRs were performed with 4 ng RNA using primers for mouse Sdc1 to -4, Gpc1, Gpc3, Gpc4, E-cadherin, fibronectin, β1 integrin, and glyceraldehyde-3-phosphate dehydrogenase (GAPDH) (see Table S1 in the supplemental material) and a Verso 1-step RT-qPCR kit on a CFX96 real-time system (Bio-Rad, Hercules, CA). Target gene expression was normalized to the housekeeping gene GAPDH using quantification cycle (Δ*C_q_*) between the target genes and GAPDH.

10.1128/mBio.01907-20.7 TABLE S1 Forward and reverse primers for qRT-PCR analyses. Download Table S1, DOCX file, 0.1 MB. Copyright © 2020 Jinno et al. 2020 Jinno et al. This content is distributed under the terms of the Creative Commons Attribution 4.0 International license.

### Histopathology.

Enucleated eyes were fixed in 4% paraformaldehyde/PBS for 4 h at room temperature, embedded in paraffin, and sectioned. Eye sections (5 μm) were deparaffinized, hydrated in xylene, 100% to 50% ethanol gradient, and PBS, and stained with hematoxylin and eosin or immunostained. For immunostaining, sections were microwaved twice in defrost mode for 6 min in 50 mM Tris, pH 8.8, and 1 mM EDTA, and background signals were quenched with 100 mM NH_4_Cl. Sections were then blocked with 10% nonimmune serum of secondary antibodies in PBS and immunostained with the indicated specific antibodies and corresponding secondary antibodies. Where primary antibodies directly conjugated to fluorophores were used, sections were blocked with 3% BSA in PBS. Images were captured with the Zeiss Axiovert 40 CFL microscope, and pictures were taken with the AxioCam MRm high-resolution camera. Adobe Photoshop CS6 was used to process the acquired images.

### Sdc1-Hep II binding interaction.

Hep II fragment was conjugated to Ultralink affinity beads to approximately 0.5 mg Hep II/ml Ultralink beads according to the manufacturer’s instructions. Purified Sdc1 (50 ng) was preincubated with PBS or increasing doses of Hep II or 45-kDa gelatin binding fragment for 30 min at room temperature, incubated overnight with 10 μl of Hep II-conjugated Ultralink beads at 4°C, and washed three times with PBS. Bound Sdc1 was eluted with 120 μl of Tris-buffered saline (TBS) containing 7 M urea and quantified by dot immunoblotting.

### Data analysis.

Data are expressed as scatterplots, bar graphs, or line graphs with mean ± standard error (SE). Statistical significance between experimental and control groups was analyzed by two-tailed unpaired Student’s *t* test and between multiple groups by analysis of variance (ANOVA) followed by Tukey’s *post hoc* test using GraphPad Prism software (version 6.0e). *P* values of <0.05 were determined to be significant.
