# Supplementary figures and images for: Syndecan-1 Promotes Streptococcus pneumoniae Corneal Infection by Facilitating the Assembly of Adhesive Fibronectin Fibrils
Source: mBio. 2020 Dec 8;11(6):e01907-20. doi: 10.1128/mBio.01907-20 (PMC7733941; doi:10.1128/mBio.01907-20)

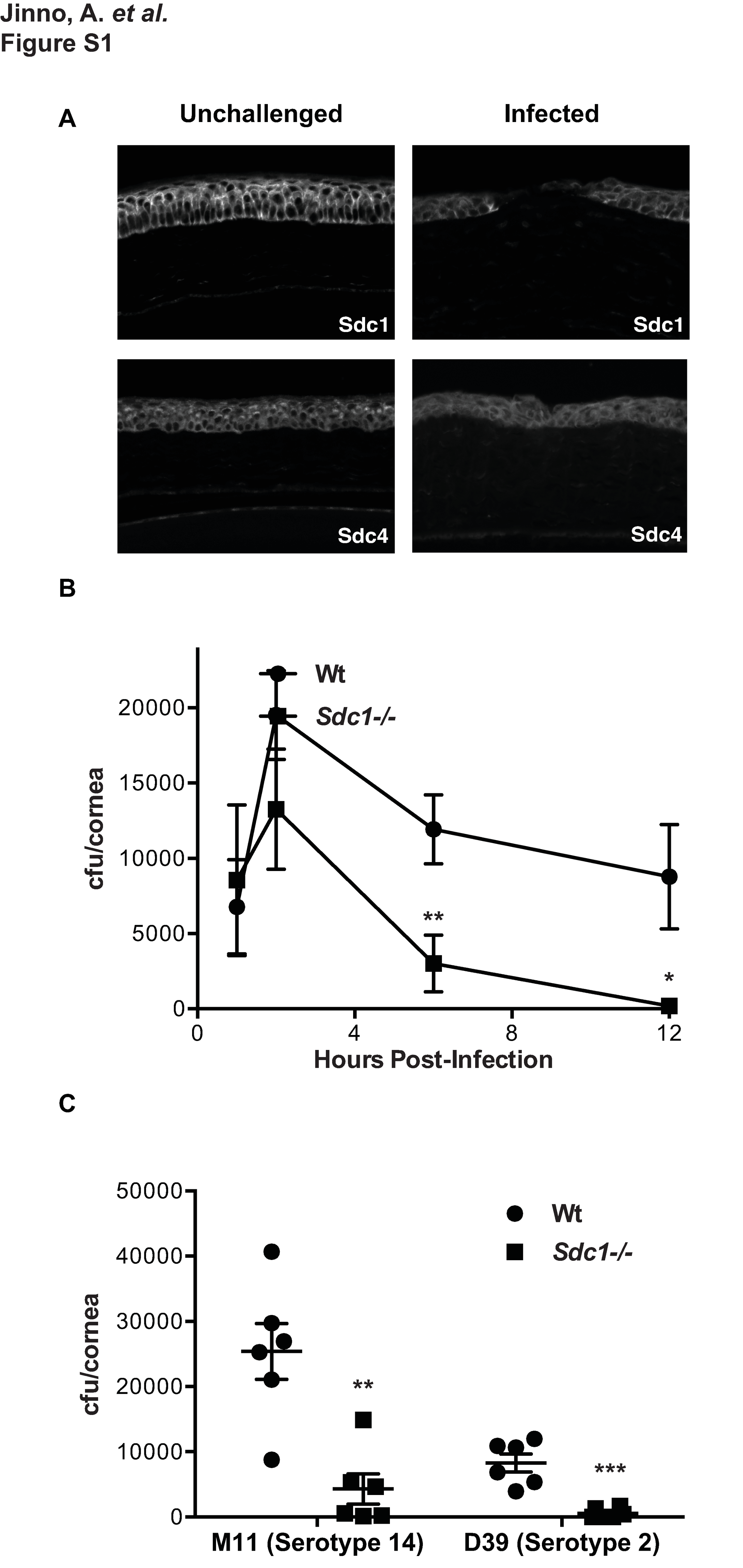

Supplement: FIG S1 [file mBio.01907-20-sf001.tif]

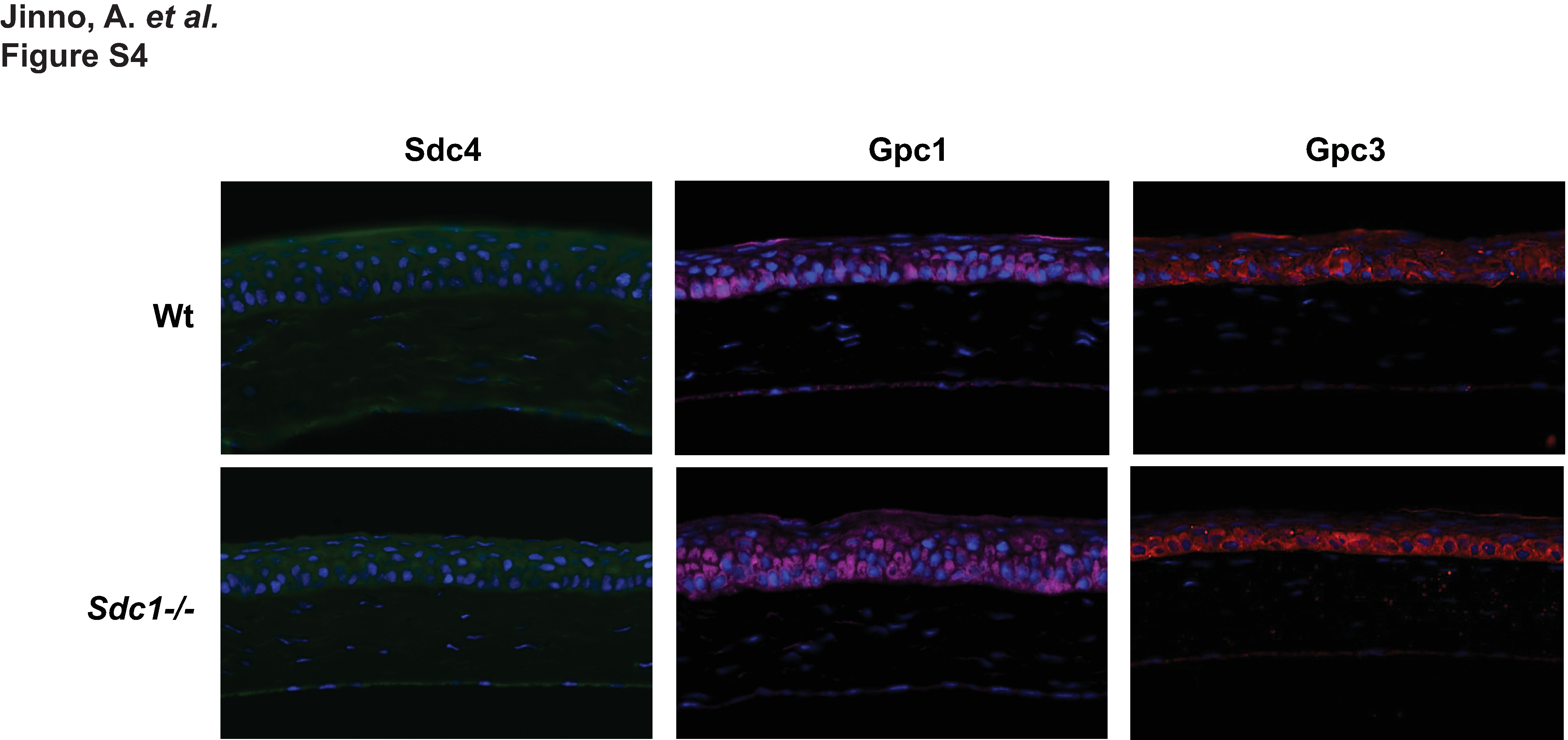

Supplement: FIG S4 [file mBio.01907-20-sf004.tif]

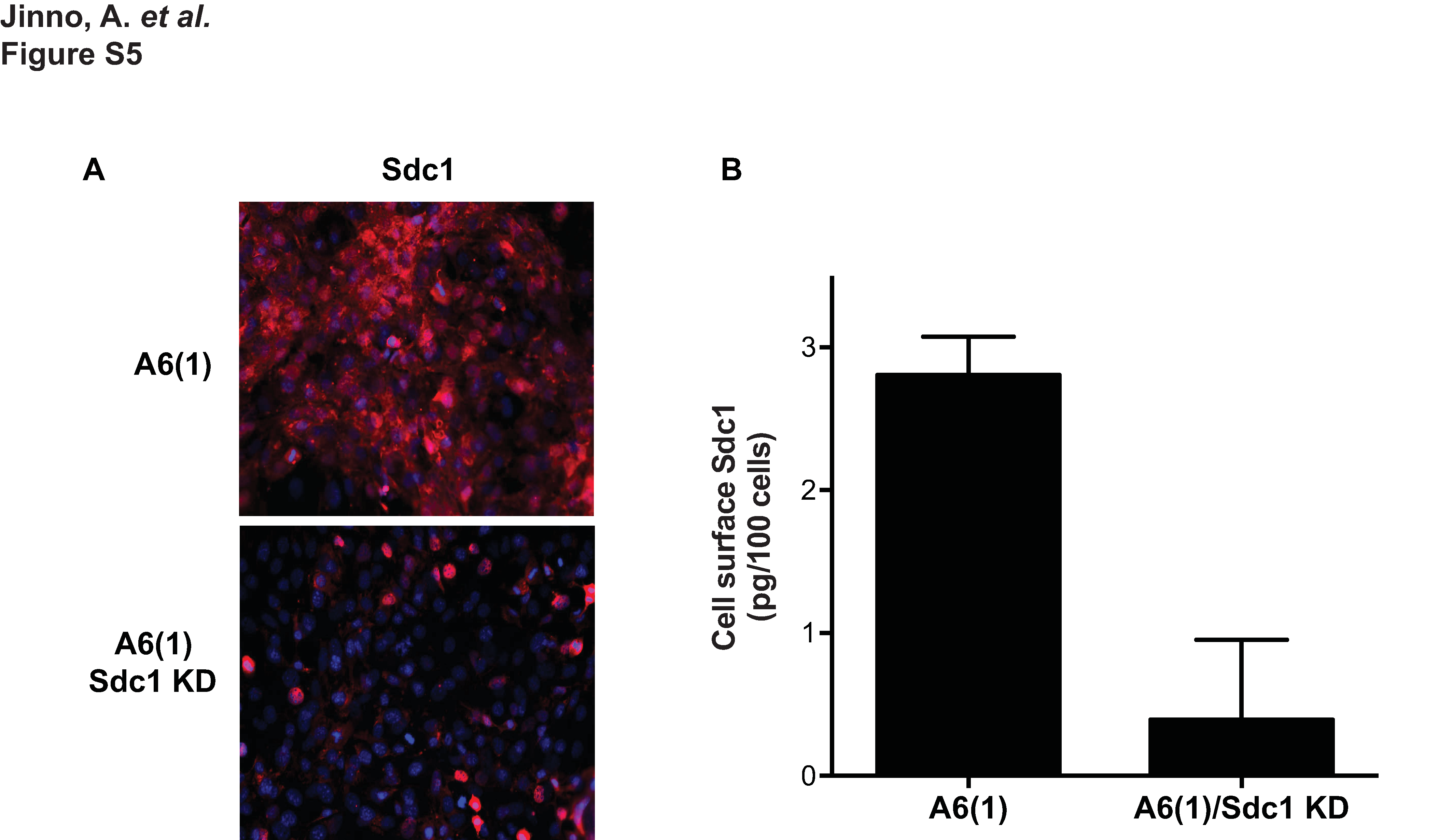

Supplement: FIG S5 [file mBio.01907-20-sf005.tif]
