# Supplementary material for: Syndecan-1 Promotes Streptococcus pneumoniae Corneal Infection by Facilitating the Assembly of Adhesive Fibronectin Fibrils
Source: mBio. 2020 Dec 8;11(6):e01907-20. doi: 10.1128/mBio.01907-20 (PMC7733941; doi:10.1128/mBio.01907-20)
Supplement: TABLE S1 [file mBio.01907-20-st001.docx]

**Table S1**

| **Mouse gene** | **Forward primer** | **Reverse primer** |
| --- | --- | --- |
| Syndecan-1 (Sdc1) | AGGATGGAACTGCCAATCAG | ATCCGGTACAGCATGAAAGC |
| Sdc2 | TCTGAGGCAGAAGAGAAGCTG | AGGATGAGGAAAATGGCAAA |
| Sdc3 | ATACTGGAGCGGAAGGAGGT | TTTCTGGTACGTGACGCTTG |
| Sdc4 | AACCACATCCCTGAGAATGC | AGGAAAACGGCAAAGAGGAT |
| Glypican-1 (Gpc1) | GGCCATCATGAAGTTGGTCT | ACACCGCCAATGACACTCTC |
| Gpc3 | TGTGCCCAAGGGTAAAGTTC | AGGTGGTGATCTCGTTGTCC |
| Gpc4 | CGTTTGCAATGATGAGAGGA | GCCATGATCTGACGAAGGAT |
| E-cadherin (Cdh1) | GAAGACAGAAACGAGACTGG | GTCTCCCTCTCAATGATGAA |
| Fibronectin (Fn1) | CCCTATCTCTGATACCGTTGTCC | TGCCGCAACTACTGTGATTCGG |
| β1 integrin (Itgb1) | CTCCAGAAGGTGGCTTTGATGC | GTGAAACCCAGCATCCGTGGAA |
| GAPDH | CATCACTGCCACCCAGAAGACTG | ATGCCAGTGAGCTTCCCGTTCAG |
